# Evaluating the impact of surgical resection on desmoid tumour growth: a retrospective analysis from the National Cancer Centre Singapore

**DOI:** 10.3389/fsurg.2026.1744644

**Published:** 2026-04-13

**Authors:** Han Yi Lee, Siqin Zhou, Si Min Chiow, Sounak Rana, Tian Ming Joshua Hoe, Ya Hwee Tan, Esther Wei Yin Chang, Valerie Shiwen Yang, Yi Ling Eileen Poon, Mingzhe Cai, Chin Jin Seo, Si Min Jolene Wong, Chin-Ann Johnny Ong, Shulyn Claramae Chia, Nagavalli Somasundaram, Mohamad Farid, Wei Ying Tham, Jason Yongsheng Chan, Jianbang Chiang

**Affiliations:** 1 Division of Medical Oncology, National Cancer Centre Singapore, Singapore, Singapore; 2 Division of Clinical Trials & Epidemiology Sciences, National Cancer Centre Singapore, Singapore, Singapore; 3 Department of Nuclear Medicine and Molecular Imaging, Singapore General Hospital, Singapore, Singapore; 4 Yong Loo Lin School of Medicine, Singapore, Singapore; 5 Division of Surgery and Surgical Oncology, National Cancer Centre Singapore, Singapore, Singapore

**Keywords:** active surveillance, desmoid, soft tissue sarcoma, surgery, resection

## Abstract

**Introduction:**

Desmoid tumours are locally aggressive, fibroblastic soft-tissue tumours which can affect adjacent structures. Management of desmoid tumours is multi-faceted and controversies surrounding surgical treatment stem from concerns about potential of surgery to precipitate tumour growth as pathogenesis of desmoid tumours is postulated to originate from trauma.

**Methods:**

Data from patients with desmoid tumours treated at the National Cancer Centre Singapore between 1999 and 2023 were collected retrospectively. We reviewed and compared radiological disease progression at one year for patients undergoing active surveillance and those who had surgical resection.

**Results:**

A total of 76 patients with desmoid tumours were seen in NCCS between September 1999 and October 2023; 19 patients were placed on active surveillance, and the remaining 57 patients underwent R0/ R1/ R2 wide excision of desmoid tumour. At one-year, progressive disease was observed in 5 out of 19 patients (26.3%) on active surveillance, and 13 out of 57 patients (22.8%) who underwent surgical resection. Progressive disease was observed in 5 out of 25 (20%) of patients following R0 resection, though no further progression occurred long-term, and all recurrences either stabilized or regressed. In the R1 group, 7 out of 28 (25%) patients showed progression at one year; management included repeat resection, cryoablation, or continued surveillance. Among R2 patients, 1 out of 4 patients had progressive disease with one patient maintaining complete radiological response at five years.

**Conclusion:**

Long-term outcomes following R0 resection were notably favourable, with no further progression and evidence of tumour stability or regression in all recurrent cases. Progressive disease rates at one year were comparable between surgical and surveillance groups, supporting a tailored, risk-adapted approach to management. Surgery does not appear to trigger tumour growth and achieves good disease control at one year, reinforcing its role as a viable option alongside active surveillance in selected patients.

## Introduction

Desmoid tumours are rare [estimated at three to five cases per million person-years (1)], locally aggressive, fibroblastic soft-tissue tumours which often affect organs and adjacent structure leading to high symptom burden (1). Desmoid tumours can occur both sporadically and in the context of hereditary cancer syndromes, such as familial adenomatous polyposis (FAP). Their clinical course is often unpredictable (2) and variable leading to difficulty in standardization of treatment. Desmoid tumours often lead to complications by exerting pressure on nearby neurovascular structures (3) or by invading into adjacent organs or bones. Patients with desmoid tumours frequently face constraints in their everyday activities due to enduring pain, reduced functioning, psychological impairment, and an overall decline in their quality of life (4). Desmoid tumours located in critical areas, such as near joints or within the abdomen, can lead to substantial morbidity, with symptoms that may include loss of limb function (5) or intestinal obstruction (6). Efforts to harmonise management strategies amongst clinicians have been made in recent years (2, 7). Retrospective analyses and a large prospective study of 771 patients by Penel et al. (8) demonstrated no significant difference in event-free survival (2-year EFS 56%) between active surveillance or surgical resection. The Dutch prospective trial (GRAFITI) cumulative incidence of the start of active treatment was 30% and PFS was 58% in patients on active surveillance (9). It is generally agreed that adopting a conservative active surveillance approach should be the primary course of action in newly diagnosed patients, unless there are pain or other clinical symptoms that warrant intervention (10). This strategy allows for a period of observation to comprehend the disease's progression and customize subsequent treatment plans accordingly. If active management is required and patients are deemed unsuitable for surgical resection, local ablative treatments such as cryotherapy or radiotherapy are options. Medical therapies such as gamma-secretase inhibitor [nirogacestat (11)], tyrosine kinase inhibitors or chemotherapy have also shown activity in control of desmoid tumour (12). Nevertheless, numerous aspects of desmoid tumour management remain ambiguous due to the notable variations observed among individual desmoid tumours (13).

Surgery remains an option for patients with desmoid tumour demonstrating rapid growth or require rapid symptom relief; however complete excision with negative margins is often not possible. The post-surgery recurrence rate is as high as 50% (14), and surgery can result in disfigurement and/or loss of function (1). The controversies surrounding desmoid tumour management also stem from concerns about the potential of surgery to precipitate further growth of the tumour (15). Desmoid tumours are postulated to originate from trauma (16). Since surgery constitutes a form of trauma characterized by inflammation and the release of growth factors crucial in wound healing, it may inadvertently stimulate the proliferation of clonal desmoid tumour cells (7). The formation of scar tissue following surgery may also create an environment that promotes tumour regrowth, especially if the wound healing process involves abnormal fibrosis. During surgery, there is also a small risk that tumour cells may inadvertently spread to nearby tissues (17). This could lead to formation of new tumours in surrounding areas, causing the disease to progress post-surgery. A cascade of local and systemic inflammatory events following surgery has been postulated to contribute to uncontrolled tumour growth, potentially through mechanisms such as the loss of inhibitory control exerted by the primary tumour after its removal (18). There is scarcity of data regarding the potential for surgery to instigate rapid growth of desmoid tumours.

Our study aimed to evaluate whether surgical management of desmoid tumours is associated with more aggressive tumour regrowth following resection. In addition, we sought to compare post-treatment outcomes between patients managed with upfront surgical resection and those managed with active surveillance. Only these two patient groups—surgery and active surveillance—were included in the present analysis.

## Methods

To address the controversies of surgery precipitating growth of desmoid tumours, we conducted a retrospective analysis of all patients who were diagnosed with desmoid tumours at the National Cancer Centre of Singapore (NCCS) over 20 years from 1999 to 2023. Serial scans were conducted to measure tumour sizes, with all radiological imaging from initial diagnosis to the last available follow-up scan being assessed by a study team member. Patients were categorized based on their desmoid tumour management: active surveillance, surgery with no residual tumour (R0), surgery with microscopic residual tumour (R1), and surgery with macroscopic residual tumour (R2). The classification of surgery was determined through analysis of surgery notes and histology reports by a study team member. Patients who were initiated on systemic therapy or radiotherapy upfront at diagnosis were excluded from analysis. Disease status at radiological investigation one year from initiation of active surveillance or one year post surgery was reviewed.

Patient demographics and clinical characteristics were summarized using descriptive statistics according to whether surgery was performed. Categorical variables were summarized as frequency and percentage, and continuous variables were summarized using median with range. Fisher exact tests were performed for categorical variables and Mann–Whitney U test were performed for continuous variables to assess the association between patient demographics and clinical characteristics with their surgery status. Percentage change in tumour size over time relative to baseline was plotted for individual patient using spider plot according to biopsy only, or R0/R1/R2 resection. Two-sided *p*-value less than 0.05 was considered statistically significant. All analyses were performed using R software (version 4.4.1).

## Results

A total of 76 patients with desmoid tumours were seen in NCCS between September 1999 and October 2023. Median age at diagnosis was 38 years old and more than half of the patients (53.9%) were diagnosed below the age of 40 years old. In the active surveillance group, most patients (78.9%) were below the age of 40 years old whilst the patients who had wide excision were older – 45.6% below 40 years old, 43.9% between 40 and 69 years old and 10.5% above the age of 70. Most patients (>70%) were female in both groups of patients (Table 1).

**Table 1:** Characteristics, procedure and disease status for patients with desmoid tumours on active surveillance vs. wide excision.

Patient characteristics	Total (*N* = 76)	Active surveillance (*N* = 19)	Wide excision (*N* = 57)	*p* value
Age				0.075#
Median (range)	38 (17–85)	32 (18–69)	41 (17–85)	
Age group				0.199
Below 40 yrs	41 (53.9%)	15 (78.9%)	26 (45.6%)	
40–49 yrs	13 (17.1%)	2 (10.5%)	11 (19.3%)	
50–59 yrs	8 (10.5%)	1 (5.3%)	7 (12.3%)	
60–69 yrs	8 (10.5%)	1 (5.3%)	7 (12.3%)	
70 yrs and above	6 (7.9%)	0 (0.0%)	6 (10.5%)	
Gender				0.765
Female	56 (73.7%)	15 (78.9%)	41 (71.9%)	
Male	20 (26.3%)	4 (21.1%)	16 (28.1%)	
Race				0.024
Chinese	54 (71.1%)	10 (52.6%)	44 (77.2%)	
Indian	8 (10.5%)	2 (10.5%)	6 (10.5%)	
Malay	9 (11.8%)	6 (31.6%)	3 (5.3%)	
Others	5 (6.6%)	1 (5.3%)	4 (7.0%)	
Sites of disease				
Abdominal wall	25 (32.9%)	9 (47.3%)	16 (28.1%)	
Intra-abdominal	15 (19.7%)	2 (10.5%)	13 (22.8%)	
Extra-abdominal	36 (47.3%)	8 (42.1%)	28 (49.1%)	
Procedure				< 0.001
Active surveillance	18 (23.7%)	19 (100.0%)	0 (0.0%)	
R0 Resection	26 (34.2%)	0 (0.0%)	25 (43.9%)	
R1 Resection	28 (36.8%)	0 (0.0%)	28 (49.1%)	
R2 Resection	4 (5.3%)	0 (0.0%)	4 (7.0%)	
Disease status at 1 year				< 0.001
Complete response (CR)	42 (55.3%)	0 (0.0%)	42 (73.7%)	
Partial response (PR)	2 (2.6%)	1 (5.3%)	1 (1.8%)	
Stable disease (SD)	14 (18.4%)	13 (68.4%)	1 (1.8%)	
Progressive disease (PD)	18 (23.7%)	5 (26.3%)	13 (22.8%)	

*P*-value estimated using Fisher's exact test unless otherwise stated.

^#^*P*-value estimated using Kruskal–Wallis test.

At one-year, progressive disease was observed in 5 out of 19 patients (26.3%) on active surveillance, and 13 out of 57 patients (22.8%) who underwent surgical resection. Surgery did not result in a significant difference in the proportion of patients with tumour growth and was not associated with an accelerated increase in tumour volume one year after surgery. Furthermore, factors such as age, gender, race, and disease sites did not appear to influence outcomes in patients with desmoid tumours.

**Figure 1:**
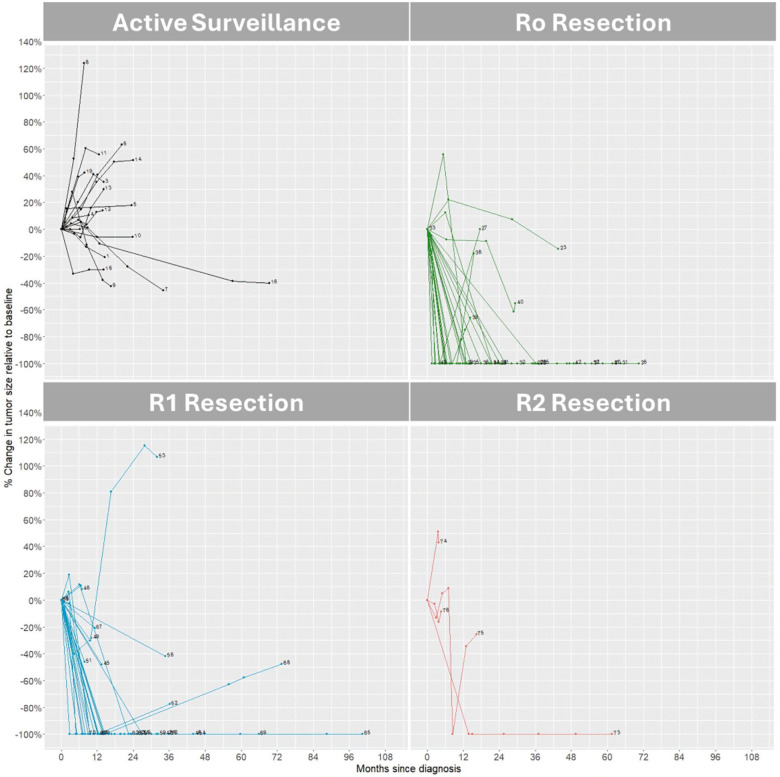
Spider-plot of percentage change in tumour size amongst four groups of patients with desmoid tumours.

Nineteen patients were managed with active surveillance. Nine patients (47.3%) had abdominal wall disease, two patients (10.5%) had intra-abdominal disease and eight patients (42.1%) with extra-abdominal disease. At one-year follow-up, none achieved a complete response with surveillance, while one patient (5.3%) had partial response without intervention. Thirteen patients (68.4%) had stable disease, accounting for most patients in the active surveillance group. Among the five patients with progressive disease, two underwent cryotherapy, one received trans-arterial chemoembolization, one underwent surgical resection, and one succumbed to extensive tumour bleeding. Twelve patients in the active surveillance group had longer-term follow-up beyond the one-year cutoff. Of these, four exhibited progressive disease, four had stable disease, and four maintained a partial response at the end of the follow-up period (Figure 1).

Fifty-seven patients underwent wide excision, sixteen patients (28.1%) had abdominal wall disease; 13 patients (22.8%) had intra-abdominal disease, 28 patients (49.1%) had extra-abdominal disease. In patients who had wide excision, 25 patients (43.9%) underwent R0 resection, 28 patients (49.1%) had R1 resection while the remaining four patients (7%) had R2 resection. At one-year post-resection, 42 patients (73.7%) maintained a complete response which represented nearly ¾ of the surgery cohort, while one patient (1.8%) exhibited partial response, and one patient (1.8%) had stable disease (Figure 1).

In the R0 resection group, at one-year follow-up, 5 out of 25 patients (20%) exhibited progressive disease. However, at longer-term follow-up, no patients showed further progression. Among the five patients who experienced tumour recurrence after R0 surgery, all tumours either underwent spontaneous regression or remained stable (Figure 1).

In the R1 resection group, 7 out of 28 patients (25%) showed progressive disease at one-year follow-up. Among these seven patients, four underwent repeat resection, one received cryoablation, and two continued active surveillance. Of the latter, one experienced significant disease progression but declined all interventions, while the other remained on surveillance at the data cutoff (Figure 1).

In the R2 resection group, consisting of 4 patients, one had progressive disease, one had stable disease, one had partial response, and once had complete radiological response at one-year follow-up. The patient with progressive disease underwent repeat resection but was lost to follow-up afterward. The patient with complete radiological response remained in complete response at five-year follow-up (Figure 1).

## Discussion

There have been significant changes in the management of desmoid tumours over the years. Historically, primary management of desmoid tumours was surgical resection. Given the increasing evidence that desmoid tumours can remain stable for years or even undergo spontaneous regression, there has been a shift towards active surveillance as a preferred approach. Despite moving towards a more conservative approach, patients with symptoms at diagnosis or those with potential significant morbidity if tumour progresses are still considered for upfront surgical resection.

In our cohort, we did not notice a significant increase in growth rate of tumours post resection in patients who underwent surgical resection. Most patients who underwent R0 or R1 resection remained in radiological complete response up to five years from resection. It is unclear if the absence of exponential growth post-surgery can be attributed to inherent tumour molecular differences or genetic variations of our Asian population. In our group of patients, many patients remained in sustained CR following surgical resection. Post-surgery recurrence of desmoid tumour was previously reported to be as high as 30%–50% in existing literature (8, 14, 19). Various factors such as younger age, extra-abdominal site, larger tumour size and close or positive margin status were associated with unfavourable outcomes (19). Our dataset consists of patients with follow-ups nearing five years, which adds strength to our conclusion. However, desmoid tumours are generally slow-growing, and recurrence rates have been reported up to ten years after surgical resection (20).

In our study, despite about half of the patients having extra-abdominal sites; the high proportion of patients with no tumour progression may be related to the high rates of R0 and R1 resection. R0 resection has been previously reported to be associated with improved outcomes (21). Furthermore, It is recognized that a microscopically positive margin after resection may not significantly affect recurrence rates (22). This is evident in our data, where both patients with R0 and R1 resection do well, with sustained CR. This may contribute as a potential reason for the excellent outcomes in our patients who underwent surgery. Surprisingly, even among the R2 resection group, most patients did not experience progressive disease, although the small sample size limits the ability to draw significant conclusions.

Among the patients undergoing active surveillance, none achieved a complete response, and only one showed a partial response without intervention at the one-year mark. During longer-term follow-up, none of the patients experienced complete tumour regression. Notably, one patient was followed for up to five years, during which the tumour size continued to gradually decrease. Studies have generally demonstrated a complete response rate of about 20%–30% in patients on active surveillance (9, 23–25). This was not seen in our study population, potentially due to the low number of study population.

Desmoid tumours are a rare condition, which makes conducting randomized studies challenging. Retrospective reviews can introduce biases, particularly as patients who undergo surgery are often selectively chosen, limiting the generalizability of the findings to the broader desmoid tumour population. This is demonstrated in our results with good CR rates for patients who underwent surgical resection. Most of the patients selected for surgical resection had tumours that were either easily accessible or posed a significant risk of morbidity if allowed to grow further, due to their size or location.

The results from our study suggest that surgical resection may provide a potential option to achieve long-term tumour control without recurrence for a large group of patients with desmoid tumours, as a significant proportion of patients achieved a complete response following surgery and for those who did not, surgery also did not precipitate growth of the tumour. Surgery remains a viable approach and could also reduce healthcare costs, as patients would require less frequent radiological evaluation compared to those on active surveillance. Additionally, upfront surgery may help prevent the progression of desmoid tumours, potentially avoiding the development of symptoms or morbidity associated with tumour growth or where future resection could become more challenging due to tumour growth.

This study has several limitations which may affect study results, including a small sample size that may affect the generalizability of the findings. Additionally, the retrospective design limits the ability to control for potential confounding factors especially as patients who underwent surgical resection are carefully selected by surgeons. Finally, the analysis with relatively short follow-up duration restricts the assessment of long-term outcomes and complications.

## Conclusion

Surgery for desmoid tumours does not appear to trigger tumour growth in our single-centre analysis and may potentially be an option to achieve prolonged tumor control for patients whose tumors are likely to be completely resected surgically. Our study supports that surgical intervention is a safe option for individuals with symptomatic desmoid tumours or those with tumours at risk of becoming symptomatic. In selected cases, surgery may also be considered for patients with progressive disease despite initial active surveillance, or when tumours pose a risk of functional compromise or local complications. Such decisions should be individualised, taking into account tumour location, anticipated surgical morbidity, patient preference, and multidisciplinary consensus. Resection can therefore be cautiously considered in these specific circumstances when clinically justified.

## Data Availability

The original contributions presented in the study are included in the article/supplementary material, further inquiries can be directed to the corresponding author.
